# 2-Deoxy-D-Glucose and its Derivatives for the COVID-19 Treatment: An Update

**DOI:** 10.3389/fphar.2022.899633

**Published:** 2022-04-12

**Authors:** Zoufang Huang, Vivek P. Chavda, Lalitkumar K. Vora, Normi Gajjar, Vasso Apostolopoulos, Nirav Shah, Zhe-Sheng Chen

**Affiliations:** ^1^ Department of Hematology, Ganzhou Key Laboratory of Hematology, The First Affiliated Hospital of Gannan Medical University, Ganzhou, China; ^2^ Department of Pharmaceutics and Pharmaceutical Technology, L M College of Pharmacy, Ahmedabad, India; ^3^ School of Pharmacy, Queen’s University Belfast, Belfast, United Kingdom; ^4^ PharmD Section, L.M. College of Pharmacy, Ahmedabad, India; ^5^ Institute for Health and Sport, Victoria University, Melbourne, VIC, Australia; ^6^ Department of Pharmaceutics, SAL Institute of Pharmacy, Ahmedabad, India; ^7^ Department of Pharmaceutical Sciences, College of Pharmacy and Health Sciences, St. John’s University, New York City, NY, United States

**Keywords:** 2-deoxy-D-glucose, 2-DG, COVID-19, coronavirus, SARS-CoV-2, glycolysis, targeted therapy

## Abstract

Treatment choices for the “severe acute respiratory syndrome‐related coronavirus‐2 (SARS‐CoV‐2)” are inadequate, having no clarity on efficacy and safety profiles. Currently, no established intervention has lowered the mortality rate in the “coronavirus disease 2019 (COVID‐19)” patients. Recently, 2-deoxy-D-glucose (2-DG) has evaluated as a polypharmacological agent for COVID-19 therapy owing to its influence on the glycolytic pathway, interaction with viral proteins, and anti-inflammatory action. In May 2020, the Indian drug regulatory authority approved 2-DG as an emergency adjunct therapy in mild to severe COVID-19 patients. Clinical studies of 2-DG corroborate that it aids in faster recovery of hospitalized patients and decreases supplemental oxygen. Herein, we describe the development process, synthesis, mechanism of viral eradication, and preclinical and clinical development of 2-DG and its derivatives as molecularly targeted therapeutics for COVID-19 treatment.

## Introduction

**FIGURE 1:**
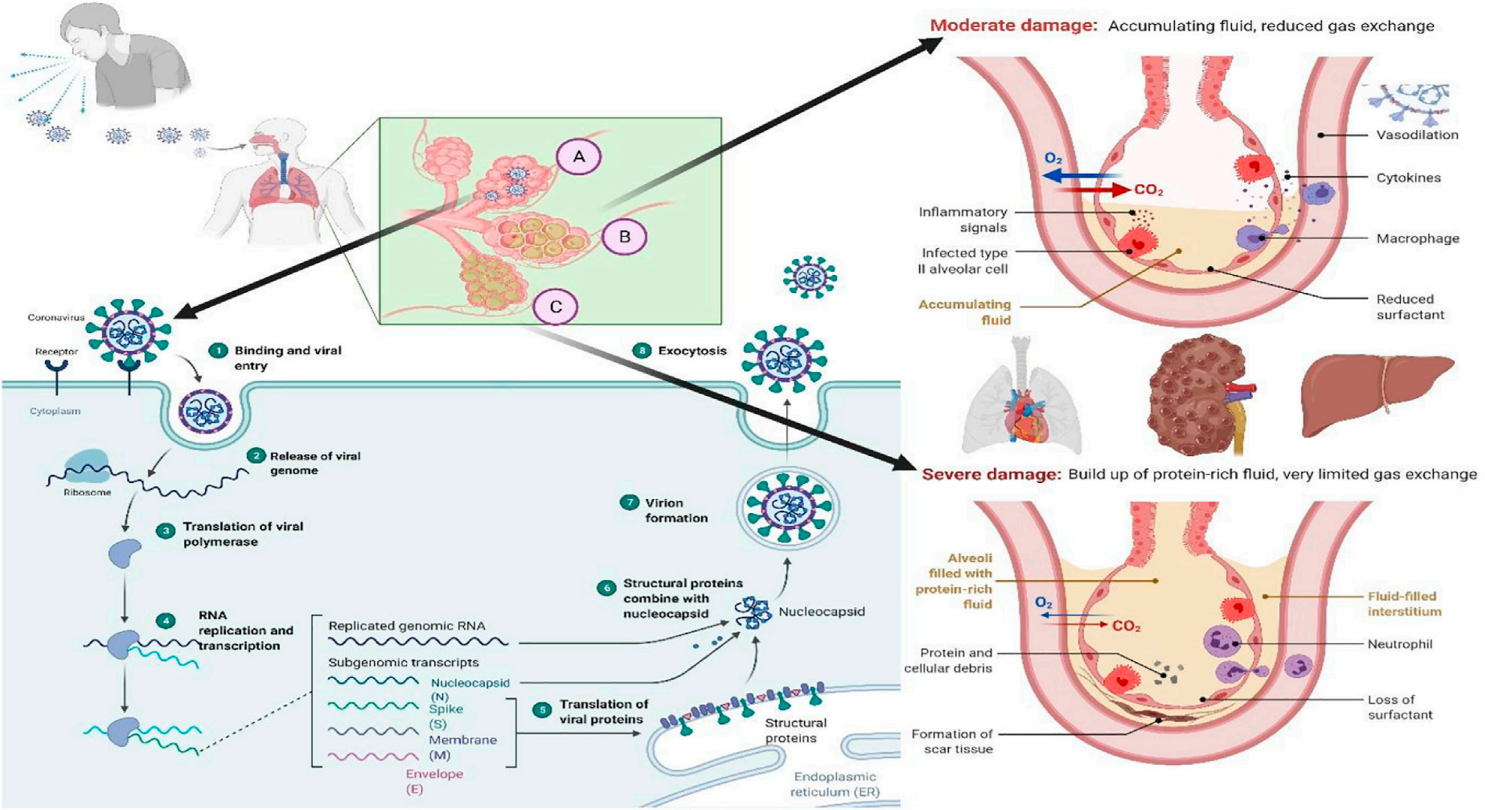
“The COVID-19 progression process in the host. (A) Viral replication in the lung epithelium: 1. Once the virus enters the respiratory tract, it binds to the ACE-2 receptor of the host cell; 2. Binding leads to the internalization of the virus inside the host cell by endocytosis and release of the viral genome; 3. Translation of viral polymerase; 4. RNA replication and transcription; 5. Translation of the viral proteins; 6. Structural proteins combined with nucleocapsid; 7. Viral assembly; and 8. Exocytosis of the virus particles. (B) As a result, the immune system attacks the infection site, killing healthy alveolar cells in the process. Reduced surfactant production by alveolar epithelial type II cells, combined with increased fluid accumulation in the alveoli, results in decreased or hindered exchange of gases. (C) Once cytokines are activated without a break, they can cause damage to the cells that react to the cytokines and halt organ function. This is referred to as a cytokine storm, and it is responsible for the transmission of severe diseases such as COVID-19”.

The perilous life-threatening COVID-19 is associated with the SARS‐CoV‐2, which was first reported to have occurred in Wuhan, China, in November 2019 (Chavda et al., 2021a). The first case was registered by the “World Health Organization (WHO)” on 31 December 2019 (Adil et al., 2021; Chavda and Apostolopoulos, 2021). The outbreak was declared a global pandemic on 11 March 2020, and as of 19 March 2022, almost 462 million individuals have been infected resulting in 6.06 million deaths globally (WHO, 2022). The progression of the COVID-19 is summarized in the Figure 1 and the readers can get the details of the same from the work of Trougakos and Colleagues (Trougakos et al., 2021) and from the work of Anant Parasher (Parasher, 2021).

Health care providers and experts around the globe are searching for appropriate COVID-19 treatment regimens (Chavda et al., 2022a; Basu et al., 2022). The emergency use approved vaccines are also not totally effective against the emerging variant of SARS-CoV-2 (Chavda et al., 2021b; Chavda et al., 2021c; Chavda et al., 2021d; Chavda et al., 2021e; Chavda and Apostolopoulos, 2022a; Chavda and Apostolopoulos, 2022b; Chavda and Apostolopoulos, 2022c). Among the massive COVID-19 cases, nearly 90% of SARS-CoV-2 infected individuals may not need hospitalization and can be treated at home, depending upon the severity of the infection. However, a small number of individuals (10%) may exhibit severe signs that necessitate hospitalization. Such cases can further complicate treatment options due to the onset of life-threatening conditions, e.g., “acute respiratory distress syndrome (ARDS)”, which may further lead to multiple organ failure (Huang et al., 2020; Chavda and Apostolopoulos, 2021). Repurposing of established drugs for the treatment of multiple diseases has recently become a popular tactic because it employs risk-free compounds with known pharmacodynamic, preclinical, and pharmacokinetic profiles that can proceed directly to the late phase of clinical trials, allowing the development of drug processes to be effectively low-cost and faster (Chavda et al., 2021f; Chavda et al., 2022a; Basu et al., 2022). Given that SARS-CoV-2 is a viral disease, a small number of anti-viral medications are available (e.g., Remdesivir, an antiviral agent that was originally employed to treat hepatitis-C), which have already been refitted into COVID-19 therapy. Nevertheless, a greater comprehension of COVID-19 pathobiology is needed to devise plans for prophylaxis and treatment. Table 1 summarizes the possible repurposed drugs for the COVID-19 management.

**TABLE 1:** Drugs repurposed for COVID-19.

Drug name	Mechanism of action	Registered Clinical trial no
Small molecule		
Carragelose nasal spray (Marinomed Biotech)	It creates a protective layer over host cell nasal mucosal membrane	NCT04590365, NCT04681001, NCT04521322, NCT04793984
Chloroquine and hydroxychloroquine tablet	Multiple pathways impede viral entry and endocytosis and host immunomodulatory effects	NCT04303507, NCT04860284
Remdesivir injection (Gilead)	Inhibits RNA polymerases (RdRps)	NCT04315948, NCT04292730
Favipiravir tablet	Inhibits RNA polymerases (RdRps)	NCT04694612, NCT04359615
Apixaban tablet (NHLBI)	Inhibit upregulated COX2 in COVID 19	NCT04498273, NCT04650087, NCT04801940, NCT04746339, NCT04512079
Icosapent ethyl tablet (Amarin Corporation)	Anti-inflammatory agent	NCT04412018, NCT04460651, NCT04505098
Abivertinib injection (Sorrento Therapeutics)	Tyrosine kinase inhibitors	NCT04440007, NCT04528667
Ivermectin tablet	Inhibiting the host importin alpha/beta-1 nuclear transport proteins	NCT04529525, NCT04438850, NCT04431466, NCT04703205
Dexamethasone tablet and injection	Anti-inflammatory agent	NCT04640168, NCT04327401,
Artesunate/pyronaridine tablet (Shin Poong Pharmaceutical Co.,Ltd.)	Act on TLR4 inflammatory signaling pathway	NCT04475107, NCT04532931, NCT04701606, NCT04695197
Hydrocortisone tablet	Anti-inflammatory agent	NCT04348305, NCT02517489
Camostat mesylate tablet (Chugai Pharmaceuticals)	Serine protease inhibitor	NCT04608266, NCT04418128, NCT04527133
Umifenovir tablet (Jieming QU)	Inhibits the viral envelope’s membrane fusion	NCT04350684
Nitazoxanide tablet	Upregulates innate immunity and act on interferon pathway	NCT04486313, NCT04359680, NCT04343248,
Losmapimod tablet (Fulcrum Therapeutics)	Reduces upregulated C-reactive protein and IL-6	NCT04511819
Bucillamine tablet (Revive Therapeutics Ltd.)	Immunomodulator	NCT04504734
Nitric oxide inhalation (Bellerophon Therapeutics)	Inhibits viral replication *via* intermediate peroxynitrite pathway	NCT04421508
Niclosamide nasal spray (Union therapeutics)	It inhibits S-Phase kinase-associated protein-2	NCT04603924, NCT04436458, NCT04749173, NCT04372082
Large molecule		
Lenzilumab injection (Catalent)	Antagonize GM-CSF signaling	NCT04583956
Canakinumab injection (Novartis)	IL-1*β* inhibitor and neutralizes IL-1*β* activity by blocking its interaction with its receptors	NCT04362813, NCT04365153
Tocilizumab injection (Genentech)	Anti-IL-6 hence supports in cytokine storm	NCT04320615, NCT04372186, NCT04409262, NCT04381936
Leronlimab injection (CytoDyn)	CCR5 antagonist which causes the downstream release of proinflammatory cytokines	NCT04343651, NCT04347239
Infliximab injection (Janssen)	TNF inhibitor	NCT04593940
Gimsilumab injection (Roivant Sciences)	GM-CSF antagonist	NCT04351243
Adalimumab injection (Abbvie)	TNF inhibitor	NCT04705844
Recombinant human plasma (BioAegis Therapeutics)	Suppress cytokine release syndrome	NCT04358406
Lanadelumab injection (Takeda)	Bradykinin release blocker	NCT04460105

Novel SARS-CoV-2 variants are causing great concern owing to their ability to resist innate or vaccine-induced immunity as well as existing therapeutics (Chavda et al., 2021g; Chavda et al., 2022a; Chavda et al., 2022b). The delta plus variant seems to have some tolerance against monoclonal antibody combination therapy, which is the only clinical distinction from the delta variant (Chavda et al., 2021b; Chavda and Apostolopoulos, 2022b). Recently, the WHO has classified a new variant of concern (VOC) as omicron, which contains more than 52 mutations, 30 of which have been discovered in the viral spike protein alone (Chavda and Apostolopoulos, 2022a; Chavda and Apostolopoulos, 2022c). In recovered COVID patients, several rare diseases including mucormycosis, white fungus infection, happy hypoxia, and other systemic disorders (multiple organ failure, immune-oncological challenges, antiphospholipid syndrome, thrombosis, etc.) have been observed (Chavda and Apostolopoulos, 2021; Iqbal et al., 2021; Nalbandian et al., 2021). In this mini review, we emphasize on the newly repurposed drug molecule 2-deoxy-D-glucose (2-DG) in the management of COVID-19.

## Targeting Glucose Metabolism for SARS-CoV-2

Even though COVID-19 and cancer are two entirely separate illnesses, the same treatment may be possible due to the similarity between the virus infected and cancer cells (Zhang et al., 2006). Viruses and cancer cells need a lot of energy as they multiply so quickly. Since glucose is the primary source of sugar in living organisms, the replicating cells consume it quickly to survive and reproduce (Fadaka et al., 2017). SARS-CoV-2 pathogenicity and replication are dependent on glycolysis and glycosylation. Glucose provides energy to viral host cells as adenosine triphosphate (ATP) derived from glycolysis, and it also allows glycan formation, which aids in the development of glycoproteins during glycosylation (Bojkova et al., 2020). Viruses change the metabolism of host cells to provide optimum conditions for fast and effective reproduction and dissemination. One important example is the increased intake of critical nutrients such as glucose to promote mitochondrial signalling, i.e., aerobic glycolysis, a dominant mechanism of glucose metabolism and its by-products for biosynthetic reactions.

**FIGURE 2:**
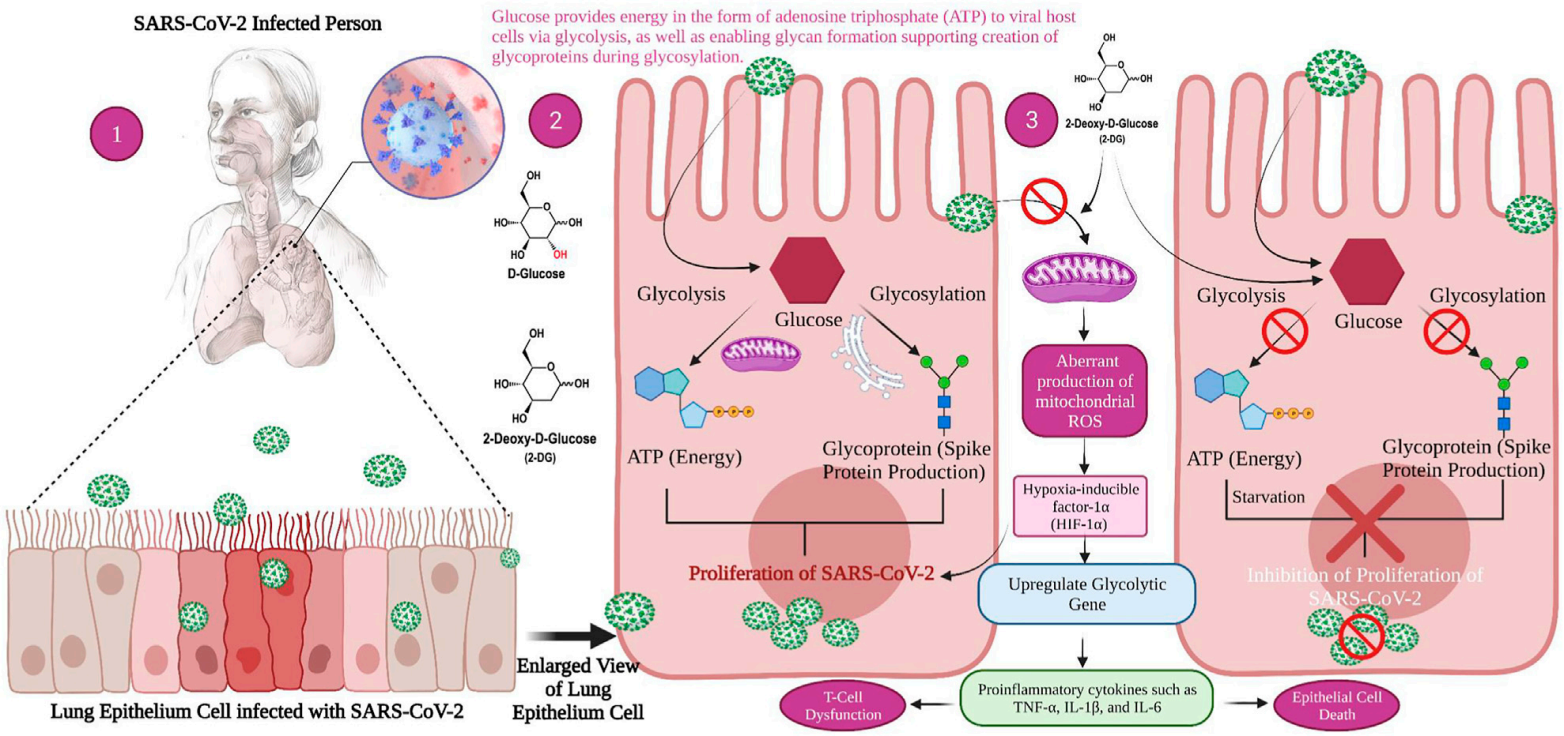
Mechanism of viral replication inhibition by 2-deoxy-D-glucose (2-DG) in SARS-CoV-2 infected host. 1. When a person is infected with SARS-CoV-2, the inflammatory action will start in the lower respiratory tract, i.e., lung epithelial cell (magnified view) 2. Glucose provides energy in the form of adenosine triphosphate (ATP) to viral host cells *via* glycolysis, as well as enabling glycan formation, thus supporting the creation of glycoproteins during glycosylation which helps the virus to replicate. 3. 2-DG as a glucose antimetabolite inhibits glycolysis and glycosylation, which ultimately inhibits viral proliferation.

Research has demonstrated that SARS-CoV-2 infection causes a metabolic resetting of human monocytes in elevated glucose cultures and promotes viral replication and cytokine formation while impairing T cell responses. The follow-up event to this causes lung epithelial cell death and, at the mechanistic level, explains why diabetic patients are at an increased threat of developing severe COVID-19 (Codo et al., 2020). Excess sugar metabolism during prolonged hyperglycemia can facilitate SARS-CoV-2 invasion and propagation, as well as an aggravated immune response. SARS-CoV-2 pathogenesis is aided by an inherent cellular approach. As a result, these cells absorb a large volume of the compound 2-DG, an antimetabolite of glucose. The 2-DG drug, like glucose, distribute across the body and enters virus-infected cells, where it stops viral synthesis and kills the protein’s energy production, preventing virus development. Since the drug inhibits both glycolysis and glycosylation, it slows virus development (Figure 2) (Sahu and Kumar, 2021).

As 2-DG has structural similarity with glucose, it binds and inhibits hexokinase - the key enzyme for glycolysis and energy production in the form of ATP. When hexokinase is inhibited, glycolysis and ATP production is suppressed, and the cell does not generate energy (Bojkova et al., 2021). Ultimately, the virus will not consume adequate energy due to the reduced energy production by the host cells, and this deprived cellular micro-environment leads to virus deactivation (Wang et al., 2015). Additionally, a reduced concentration of ATP activates the AMP (adenosine monophosphate) mediated protein kinase, followed by phosphorylation of mTOR kinase (the mechanistic target of rapamycin kinase) and tuberous sclerosis (TSC 1) (Aft et al., 2002). Thus, autophagy of virus-infected cells and cell death occurs because of the inhibition of the G1 phase in the cell cycle (Sottnik et al., 2011). Similarly, 2-DG inhibits SARS-CoV-2 multiplication in Caco-2 colon cancer cells, a common model used for the intestinal epithelial barrier (Bojkova et al., 2020).

## Glucose Analogues for SARS-CoV-2 Treatment

### 2-Deoxy-D-Glucose

**FIGURE 3:**
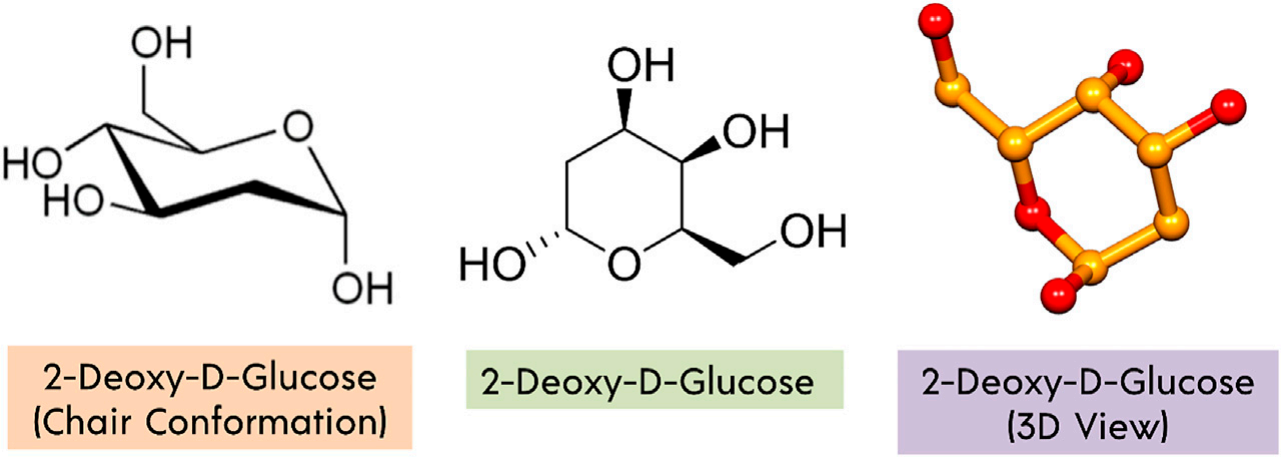
The structure of 2-deoxy-D-glucose.

2-DG (Figure 3) is a glucose molecule with hydrogen swapped for the 2-hydroxyl bonds, restricting further glycolysis. It functions at the level of phosphoglucoisomerase to impede the synthesis of glucose-6-phosphate from glucose (step 2 of glycolysis) (Wick et al., 1957). In the liver and kidneys, glucose hexokinase phosphorylates 2-DG, capturing the compound 2-deoxyglucose-6-phosphate intracellularly; therefore, coded forms of 2-DG provide a strong indication of tissue glucose absorption as well as hexokinase operation. Glucose uptake and hexokinase levels are elevated in many cancers.

2-DG is one investigational drug that is being tested for the treatment of cancer and viral infections (Aft et al., 2002). It was utilized in combination with chemotherapy and radiotherapy for treating solid tumors. However, the use of 2-DG as an individual therapeutic agent involves certain risks as it may cause toxicity to the individual by continuous administrations of high 2-DG doses over a long time. In addition, a single therapeutic dose of the drug has been noted to cause glucocytopenia in the nervous system, and irregularities in the cardiovascular, respiratory, and immunological systems as well as some normal tissues (Dwarakanath and Jain, 2009).

2-DG inhibits glycolysis, thereby creating depletion of energy and ultimately hampers cell growth (Ralser et al., 2008). 2-Deoxy-D-glucose is able to prevent N-glycosylation and other processes in mammalian cells due to its structural resemblance to mannose, causing endoplasmic reticulum stress and activating the unfolded protein response (UPR) pathway (Kurtoglu et al., 2007; Xi et al., 2011; Defenouillère et al., 2018). In addition, 2-DG can be bio-transformed *via* the pentose phosphate pathway, at least in red blood cells (RBCs), but its implications related to other cell types as well in cancer cells are unknown. The ketogenic diet has been studied for epilepsy therapy, and the importance of glycolysis in epilepsy was evaluated, and the diet has shown promise as an anti-epileptic agent (Garriga-Canut et al., 2006). It is useful in cancer diagnostics for positron emission tomography (PET), owing to the increase metabolism, 2-DG is selectively taken up by tumour cells in an excessive amount. The radioactively labeled 2-DG causes these cancer cells to glow brightly, allowing them to be seen in imaging (Sahu and Kumar, 2021).

### Molecular Docking and Synthesis Process

**FIGURE 4:**
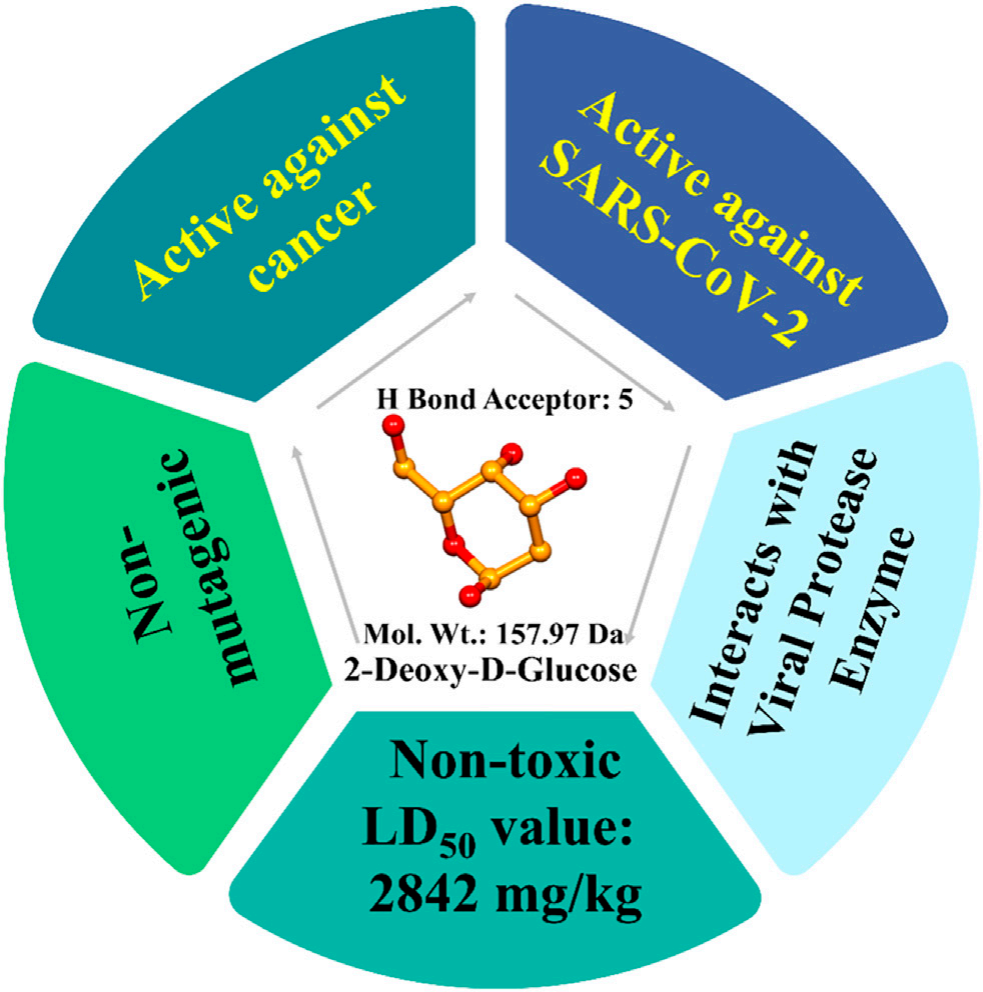
Summary of 2-Deoxy-D-glycose molecular properties and docking outcomes.

Molecular docking is often used to predict preferred orientations of one molecule in relation to another molecule and binding conformations of small drug ligands to a desired binding site or receptor (Lengauer and Rarey, 1996). Using such *in silico* methods, SARS-CoV-2 viral nuclease nsp15 endoribonuclease (PDB ID: 6VWW), main protease or 3CLpro (PDB ID: 1Q2W), and the spike glycoprotein (PDB ID: 6VSB) were used to determine interactions of the drugs lopinavir, favipiravir and hydroxychloroquine (Balkrishna et al., 2020). It was noted that 2-DG had a binding energy −140.05 kcal/mol with 3CLpro, which was superior to lopinavir (−124.00 kcal/mol). Similarly, the binding energy with endonuclease was −168.65 kcal/mol, which is superior to the drug favipiravir (−128.00 kcal/mol) and -118.31 kcal/mol with the spike glycoprotein. In addition, a derivative of 2-DG, namely 1, 3, 4, 6-Tetra-O-acetyl-2-deoxy-D-glucopyranose, was shown to have superior binding energies compared with lopinavir, favipiravir, and hydroxychloroquine. Further, 2-DG follows all Lipinski’s parameters as molecular properties of a drug in regards to bioavailability and drug likeliness (Lipinski, 2004). Bioactivity analysis and toxicity studies suggest that the 2-DG and its derivative can produce sufficient oral bioavailability without any major side effects or toxicity (Balkrishna et al., 2020). The drug has 379 mg/ml water solubility and -2 LogP value (Figure 4) Drugbank (2013).

**FIGURE 5:**
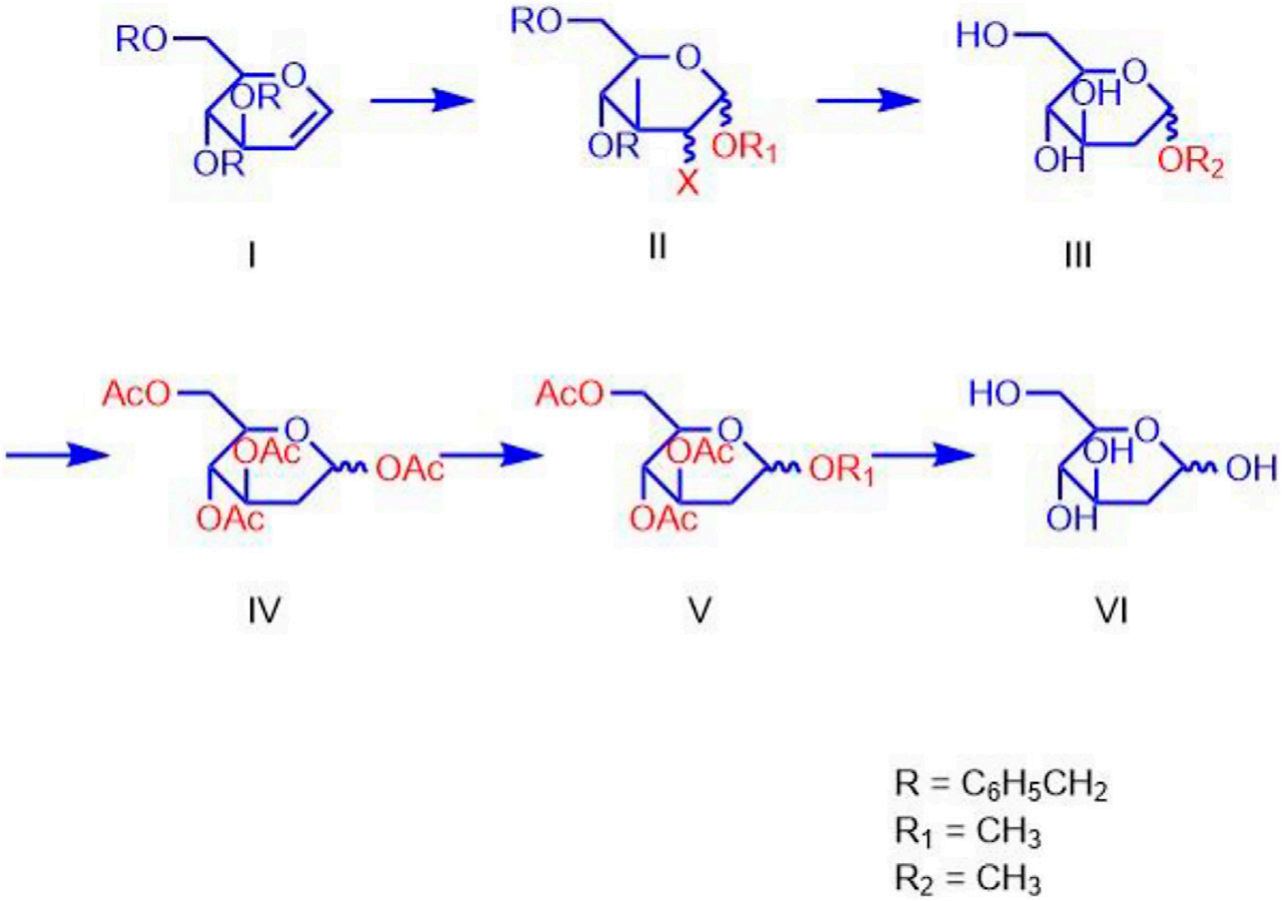
Chemical Synthesis steps for the 2-deoxy-D-glucose.

For synthesizing 2-DG, Mereyala et al. performed haloalkoxylation of R-D-Glucal, where R = H and 3, 4, 6-tri-O-benzyl. They obtained alkyl 2-deoxy-2-halo-R-*α*/*β*-D-gluco/mannopyranoside, which was further converted into alkyl 2-deoxy-2-halo-R-*α*/*β*-D-gluco/mannopyranoside by reduction to alkyl 2-deoxy-*α*/*β*-D-glucopyranoside followed by hydrolyzation to alkyl 2-deoxy-*α*/*β*-D-glucopyranoside and to 2-DG (Figure 5) (Mereyala and Kumar, 2014).

### Preclinical and Clinical Development

2-DG is consumed by the SARS-CoV-2 contaminated cells. However, it is unclear whether 2-DG has the ability to destroy or damage normal cells or whether it has any effect at all on them. When weighing costs and advantages, the most critical question is whether the benefits outweigh the risks. On critical analysis, it can be stated that there are demonstrated benefits of 2-DG and that they outweigh the risks; hence, it is currently being provided to individuals with mild to severe COVID-19 infection. These patients have a significantly better chance of survival treated with 2-DG than people who receive traditional medications (The Indian Express, 2021).

**FIGURE 6:**
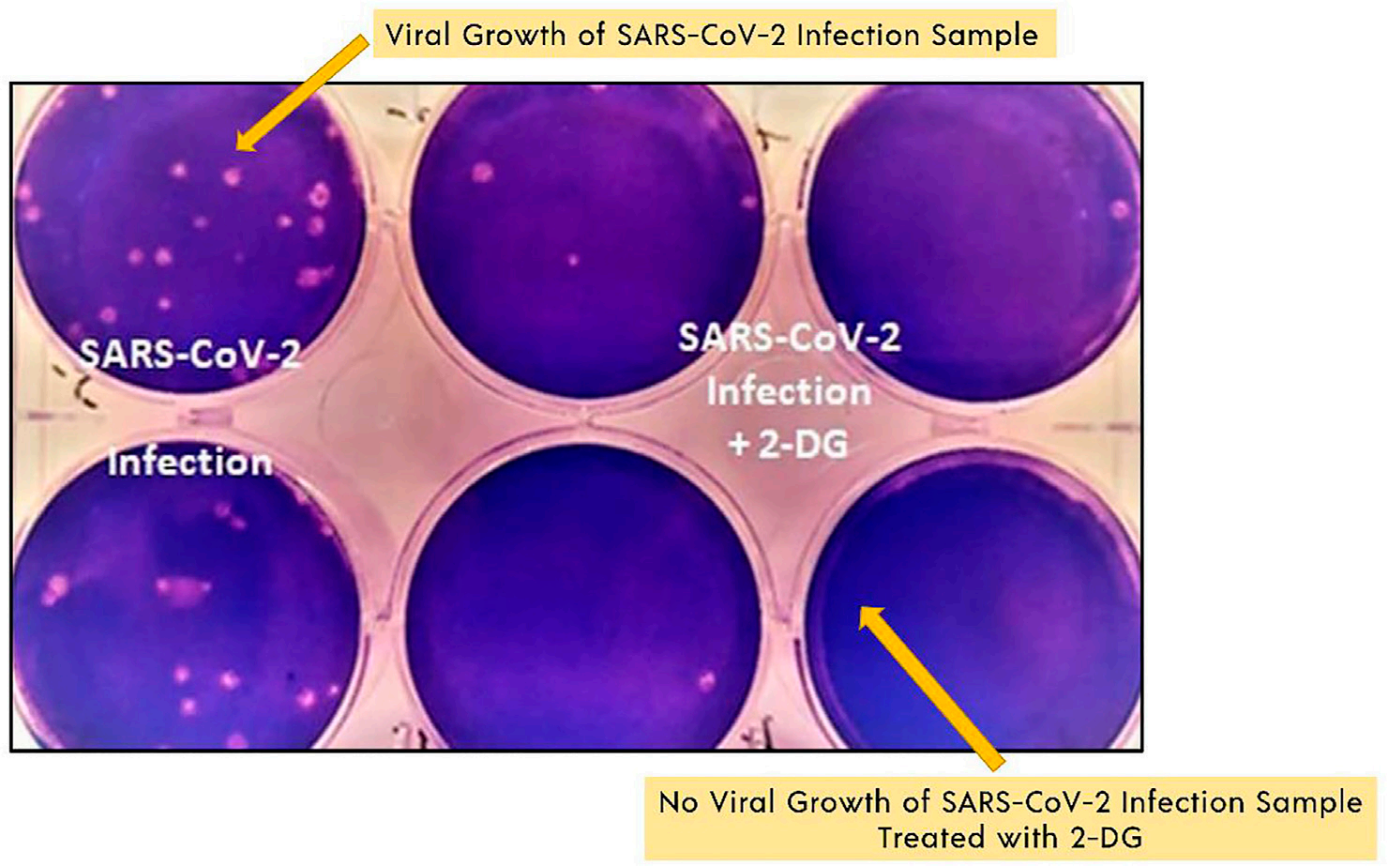
SARS-COV-2 growth inhibition study results with 2-DG. Image reproduced from the Indian government website (Ministry of Defence, 2021a).

“Defence Research and Development Organisation (DRDO)” took the lead in designing an anti-COVID-19 medicinal implementation of 2-DG. INMAS-DRDO scientists, with the assistance of the “Centre for Cellular and Molecular Biology (CCMB)”, Hyderabad, performed diffrent lab experiments during the first wave of COVID-19 pandemic in April 2020 that showed 2-DG was effective against the SARS-CoV-2, inhibiting viral development (Business Standard, 2021). “Drug Controller General of India and Central Drugs Standard Control Organization approved Phase-II clinical trials of 2-DG in COVID-19 patients in May 2020. DRDO and its business partner, Dr Reddy’s laboratories, have begun clinical trials to assess safety and effectiveness of 2-DG in COVID-19 patients (Figure 6) (The Indian Express, 2021)”.

2-DG is stable in patients with COVID-19 and shows substantial progress in their rehabilitation in phase II clinical trials (including dose-ranging) performed from May to October 2020 (Ministry of Defence, 2021a; Ministry of Defence, 2021b). Phase IIa human clinical trials were conducted in 6 hospitals, whereas phase IIb clinical trials (dose-ranging) were conducted in 11 hospitals worldwide. A phase II study was carried out in 110 COVID-19 patients. In terms of effectiveness, those who were given 2-DG had a quicker symptomatic relief compared to those treated with standard of care (SOC) on a variety of endpoints (Ministry of Defence, 2021a; Ministry of Defence, 2021b). Comparing the median time for achieving normalization of basic vital sign factors to the SOC, a marginally favourable pattern (2.5 days difference) was observed. Based on the positive outcomes, DCGI approved phase III clinical trials in November 2020.

The phase III human clinical trial in 220 patients was conducted between December 2020 and March 2021 at 27 COVID-19 hospitals in the states of Uttar Pradesh, Delhi, Gujarat, West Bengal, Maharashtra, Rajasthan, Telangana, Andhra Pradesh, Tamil Nadu, and Karnataka. In comparison to SOC, a greater proportion of patients with in 2-DG arm proceeded symptomatically and many were free of mechanical ventilation reliance by day 3 (42 percent vs. 31 percent). An identical pattern was noted in the case of patients over the age of 65 (The Indian Express, 2021). Various potential side effects of 2 -DG are reversible hyperglycemia, gastrointestinal bleeding, and QTc prolongation (Raez et al., 2013). Hyperglycemia may also worsen, particularly as the majority of COVID-19 patients are also on large dosages of steroids (Shah and Goel, 2021). The major loophole of the current phase II (*n* = 110) and Phase III (*n* = 220) clinical trial study data is very limited study population. Individuals with multi-organ dysfunction, ARDS, and those on mechanical ventilation were also omitted from the trial. Individuals with any type of chronic comorbidity were also barred from participating in the trial.

## Theranostic Role of 2-DG in Management of Cytokine Storm in COVID-19

2-DG has also proven to be a polypharmacological agent for COVID-19 therapy, due to its role in the glycolytic pathway, interaction with viral proteins, and anti-inflammatory activity (Dwarakanath and Jain, 2009; Thaker et al., 2019). Verma et al. conjectured that 2-DG could enhance the efficacy of low-dose radiation therapy (LDRT) in the treatment of COVID-19 pneumonia (Verma et al., 2020). Furthermore, the azido analogue of 2-DG, 2-azido-2-DG, has the ability to trigger catastrophic oxidative stress in a short time in severely ill COVID-19 patients (Verma et al., 2020).

**FIGURE 7:**
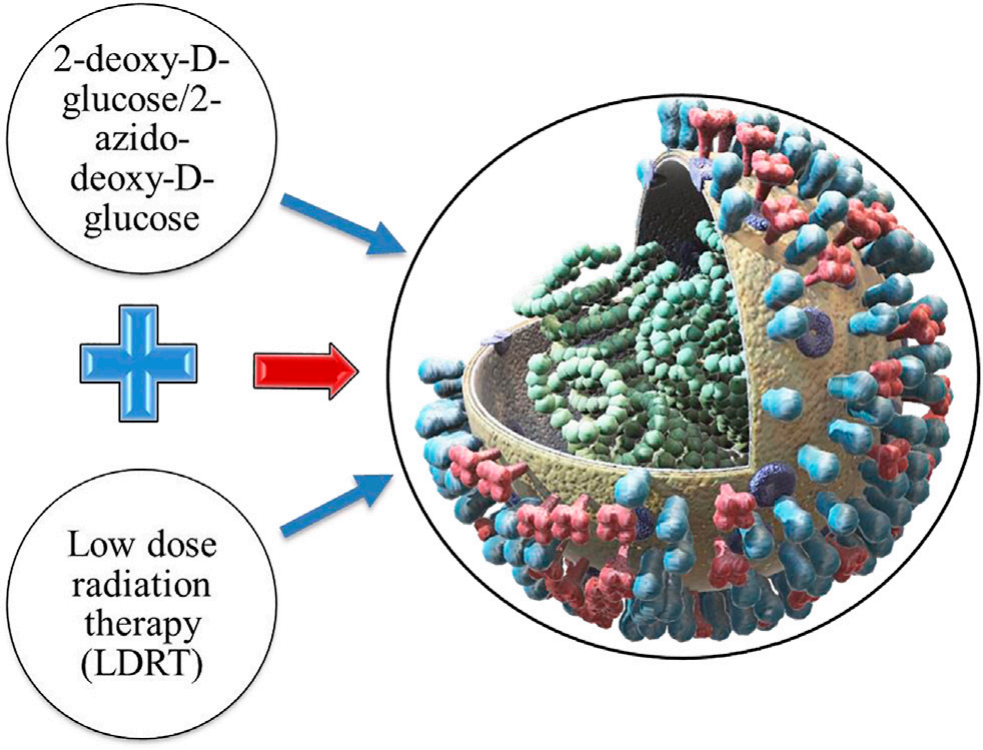
2-DG/2Azido-2-DG based radiation therapy in SARS-CoV-2 eradication.

Low dose radiation therapy can decrease the cytokine storm in COVID-19 patients owing to its capability to induce anti-inflammatory responses. Its pro-inflammatory assisted immune responses in patients infected with SARS-CoV-2 has been advocated to be an efficacious option for COVID-19 pneumonia therapy (Figure 7). Although, the timing of LDRT treatment is the most crucial factor as it may influence moderate versus severe conditions differentially. Appropriately, numerous LDRT protocols for COVID-19 patients are being evaluated with promising preliminary outcomes (Rödel et al., 2020; Wilson et al., 2020).

Because of the polypharmacological effects of 2-DG on infected lung cells, 2-DG could be a potential adjuvant to LDRT as it suppresses viral replication and thereby avoids damage to the lungs. It primarily includes suppressing the glycolysis (thus the energy status), changes in glycosylation of viral proteins, and regulation of inflammatory responses (cytokine storm). The combination can shield other vulnerable organs and tissues with subsequent reduction of death rate (Papineni et al., 2018). Still, rigorous studies on the optimization of dose and dosing frequency, as well as the assessment of associated toxicities, are needed.

### 2-Deoxy-D-Glucose Prodrug (WP1122)

**FIGURE 8:**
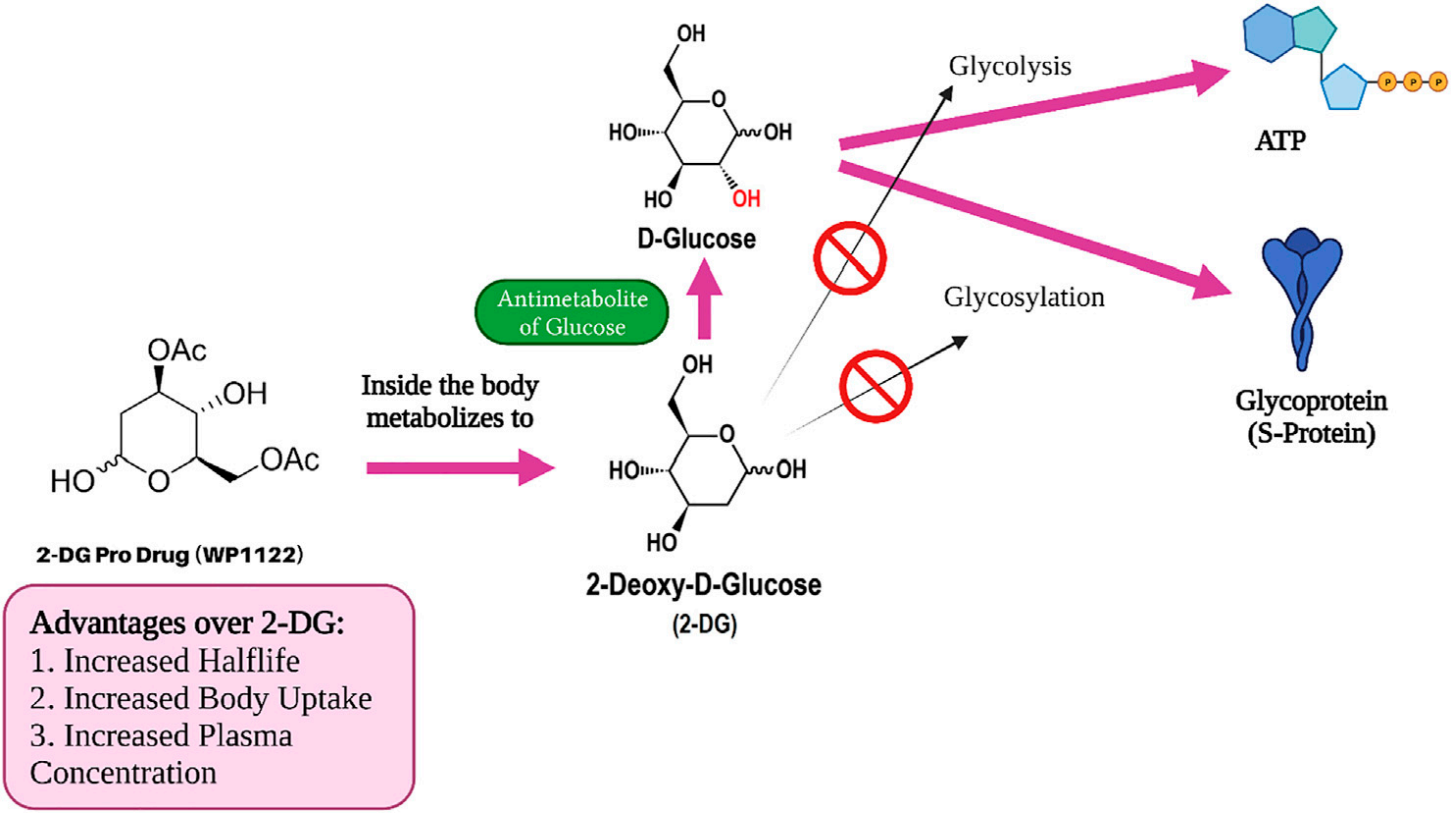
2-DG Prodrug and its role in inhibition of viral replication.

Moleculin Biotech Inc., in collaboration with Texas University (United States), has synthesized and evaluated a 2-DG prodrug candidate, WP1122, and its analogs for COVID-19 treatment. The main reason behind using this prodrug approach was that 2-DG does not possess “drug-like properties” (Pajak et al., 2019). It metabolizes quickly (has a short half-life) and fails to provide the required concentration levels in organs and tissues. Principally, the achievement of sufficient concentration of 2-DG inside target organs and tissues to inhibit viral replication has not been possible in patients (Moleculin, 2021). Hence, WP112 with improved drug-like properties (Figure 8) is a promising analogue of 2-DG as a new anti-cancer agent, in addition to improving those with COVID-19.

This chemically modified 2-DG is transformed into pharmacologically active 2-DG in the body following administration (Figure 7). The scientists have already proven *in vivo* that WP1122 reaches a much higher concentration in tissues and organs compared to 2-DG alone. Interestingly, the level after WP1122 administration is nearly twice as much its half-life while the peak plasma concentration increases by almost three times. When 2-DG is delivered *via* WP1122, it provides a much higher concentration of 2-DG in the organs and tissues *in vivo*, as compared to when it is given directly (Zielinski, 2017). However, the greatest improvement seen is in the organ uptake and its potency. Moleculin Biotech’s previous work using WP1122 in cancer, even before the arrival of COVID-19, showed that WP1122 was 10 times more potent than equimolar doses of 2-DG alone. Recently, they have planned a clinical trial of WP1122 for COVID-19 (Stein et al., 2010; Zielinski, 2017).

## Conclusion

Targeting glucose metabolism may offer new practical antiviral approaches for the treatment of mild to severe SARS-CoV-2 infection, especially in individuals with metabolic diseases. The 2-DG was designed to treat critically ill patients with COVID-19 in recent clinical trials in India. The Indian drug authority approved 2-DG as emergency use, which is available in powder form in sachets for oral route administration as an adjunct therapy. It builds up in virus-infected cells, prevents viral synthesis and energy output, and thereby inhibits viral replication. Its selective aggregation in the infected cells distinguishes this compound. Another promising approach is to use the combination of 2-DG and its derivative agents with different treatment modalities to inhibit the life cycle of the virus. This may slow down drug resistance development and decrease the effective concentration of individual drugs, reducing possible adverse effects. The findings are premature, and the individuals who will get 2-DG will need to be closely monitored. The creation of an online data repository to record the efficacy and adverse effects of 2-DG would be extremely beneficial in integrating data on 2-DG usage in COVID-19 illness. A multicentric investigation with a bigger population and from diverse geographical locations is needed to corroborate the preliminary results.
